# Tracking two pleasures

**DOI:** 10.3758/s13423-019-01695-6

**Published:** 2020-01-02

**Authors:** Aenne A. Brielmann, Denis G. Pelli

**Affiliations:** 1 Psychology Department, New York University, New York, NY USA; 2 Center for Neural Science, New York University, New York, NY USA

**Keywords:** Pleasure, Ensemble perception, Aesthetics, Glimpse

## Abstract

Can people track several pleasures? In everyday life, pleasing stimuli rarely appear in isolation. Yet, experiments on aesthetic pleasure usually present only one image at a time. Here, we ask whether people can reliably report the pleasure of either of two images seen in a single glimpse. Participants (*N* = 13 in the original; +25 in the preregistered replication) viewed 36 Open Affective Standardized Image Set (OASIS) images that span the entire range of pleasure and beauty. On each trial, the observer saw two images, side by side, for 200 ms. An arrow cue pointed, randomly, left, right, or bidirectionally. Left or right indicated which image (the target) to rate while ignoring the other (the distractor); bidirectional requested rating the combined pleasure of both images. In half the blocks, the cue came before the images (precuing). Otherwise, it came after (postcuing). Precuing allowed the observer to ignore the distractor, while postcuing demanded tracking both images. Finally, we obtained single-pleasure ratings for each image shown alone. Our replication confirms the original study. People have unbiased access to their felt pleasure from each image and the average of both. Furthermore, the variance of the observer’s report is similar whether reporting the pleasure of one image or the average pleasure of two. The undiminished variance for reports of the average pleasure of two images indicates either that the underlying pleasure variances are highly correlated, or, more likely, that the variance arises in the common reporting process. In brief, observers can faithfully track at least two visual pleasures.

**Electronic supplementary material:**

The online version of this article (10.3758/s13423-019-01695-6) contains supplementary material, which is available to authorized users.

## Introduction

How often have you relied on online ratings to choose a restaurant? Such ratings typically provide not only a summary score but also break down the rating into several dimensions. Zagat, for instance, gives ratings on food, decor, and service. But are these subscores independently informative, or just a noisy reflection of the overall rating? After all, the rater smelled and tasted the food as she listened to the ambient music and saw the room’s decor. Can anyone disentangle each of these pleasures from the rest?

Past attempts to generalize from experiments to daily life have implicitly assumed that everyone can distinguish one pleasure from other simultaneous pleasures. Following the reductionist approach of science, the experiments typically analyze the perception of one isolated stimulus. In experimental aesthetics, for instance, the prototypical study shows one image at a time and observers report how beautiful or pleasing they find it (e.g., Cotter, Silvia, Bertamini, Palumbo, & Vartanian, 2017; Graf & Landwehr, 2017; Marin & Leder, 2016; Nakamura & Kawabata, 2015; Savazzi et al., 2014). In everyday life, however, we enjoy the pleasantness of any kind of object (e.g., the attractiveness of a face, the taste of popcorn, the sound of music) in the context of others (e.g., other faces in a crowd, the movie theater, street noise). Thus, to apply any understanding of the processing of an isolated stimulus to daily life, we must ask whether the processing of one stimulus is affected by the processing of others.

### Reporting from one item in an array: compulsory averaging

When people are shown several stimuli at once and then asked about a property of one, like size or color, their reports are biased toward the mean of the set (Brady & Alvarez, 2011; Haberman & Whitney, 2009; Maule, Witzel, & Franklin, 2014). For tight arrays in peripheral vision, this kind of averaging is not merely possible, but compulsory (Parkes, Lund, Angelucci, Solomon, & Morgan, 2001). Haberman and Whitney (2009) found that observers cannot faithfully remember the individual emotions of a set of simultaneously presented faces. Rather, their responses were biased toward remembering the average emotion of the entire array of four to 16 faces. Their findings could not be explained by supposing that observers simply make noisy measurements of each individual face’s emotion. When estimating the size of a single dot, observers’ estimates are biased toward the average size of all presented dots (Brady & Alvarez, 2011; Corbett, 2017). People are also biased to mistake the average color of two simultaneously presented color patches as being one of the originally presented colors (Maule et al., 2014). These results suggest that people have limited capacity for parallel encoding and storage of simultaneously presented visual stimuli. This has been shown for both high-level and low-level features (low: size, color; high: faces’ emotional expression).

The above studies asked participants to report a stimulus feature. To our knowledge, no study so far has investigated how well people can selectively report the subjective feeling an individual stimulus elicits when presented among other stimuli. The current study aims to close this gap by asking whether people can report the felt pleasure of each of two simultaneously presented images.

### Reporting the average from an array of items: ensemble coding

To better discover whether people cannot help but average the pleasure of simultaneously displayed images, we also assess whether people can accurately average pleasure when asked to do so. Several studies show that people can make unbiased reports of the average feature value for a set of items shown on a single display. This is true for *low*-level visual features like orientation (e.g., Parkes et al., 2001), size (de Fockert & Marchant, 2008), position (Alvarez & Oliva, 2008), motion (e.g., Watamaniuk, 1993), and number (e.g., Burr & Ross, 2008) as well as *high*-level features such as facial identity (e.g., Neumann, Schweinberger, & Burton, 2013) and emotion (Fischer & Whitney, 2011; Haberman & Whitney, 2007).

Since averaging across several independently noisy estimates would reduce noise, one might expect ratings of averages to be more reliable than single-item reports. However, studies have repeatedly shown that the precision (1/*SD*) of reports of averages is conserved across different set sizes (e.g., Ariely, 2001; Chong & Treisman, 2005) and no higher than for single items (Allik, Toom, Raidvee, Averin, & Kreegipuu, 2013). The finding that increased set size fails to decrease the variability of average reports suggests that the variance of reporting is limited by a late noise that arises after combining the members of the ensemble.

Yet, we do not yet know whether observers accurately average the pleasure of two images when judging both. Two studies on high-level features found systematic deviations from averaging. One found that, when judging the average expression of a set of faces, observers discount the emotional expression of outlier faces whose emotional expression is far removed from the average expression of the rest (Haberman & Whitney, 2010). The authors thus observed a compression of observers’ ratings; observers reduced the weight of extreme values. A second study showed that observers rated the lifelikeness of the least lifelike ensembles as even less lifelike than predicted based on the averaged single-image ratings and rated the most lifelike ensembles as more lifelike than predicted (Leib, Kosovicheva, & Whitney, 2016). In this case, ratings were expanded; average ratings were lower at the low end and higher at the high end than predicted based on the arithmetic average of single-image ratings.

It remains unclear how accurately people can report the average of features other than low-level stimulus properties. Studies of ensemble perception have so far focused on stimulus properties rather than feelings provoked by the stimuli. We still do not know whether people can access subjective feelings across an array of items. If so, the averaging question becomes whether the report of the average feeling matches the average of feelings reported for each item individually and how the variance of the reported average compares with that predicted by averaging.

### Current study

We here take a minimal approach and ask (1) Can observers faithfully retain knowledge of the pleasure of *each* of two simultaneously presented images? (2) Can observers faithfully report the *average* pleasure of two simultaneously presented images? Based on the literature, we consider three alternative models as a possible answer to each question. (1) When asked to report the pleasure of one of two simultaneous images, observers could either (a) faithfully report the pleasure of one image (*faithful*) or (b) always average pleasure across both presented images (*compulsory averaging*), or (c) partially bias their pleasure report toward the pleasure of the irrelevant image (*partial compulsory averaging*). (2) When reporting the combined pleasure from both simultaneously presented images, observers could either (a) faithfully average the pleasures of both images (*faithful*) or (b) reduce the weight of images with extreme pleasure values (*compressive*), or (c) make average pleasure ratings that are lower at the low end and higher at the high end than predicted based on the ratings for the images presented in isolation (*expansive*).

## Method

The data reported here are a preregistered replication (https://osf.io/x9wsf/) of an original study conducted in 2017. The methods of the replication were identical to those of the original. The results of the replication (see Results section) confirm those of the original study (in Supplementary Results Pilot Study).

### Participants

Observers in the current study were 25 undergraduate students at NYU. We did not record the age or gender of our participants because we had no hypotheses relating to this information and collected only necessary personal information in line with the ethics board guidelines. All participants were 18 years or older. They were recruited from the NYU student body where 80% of students are female, and the average age is 21.1 years (*SD* = 1.3 years). All participants gave written informed consent according to the declaration of Helsinki. Approval was obtained from the NYU University Committee on Activities Involving Human Subjects (UCAIHS; IRB-FY2016-404). Each participant received course credit as compensation.

### Stimuli

We selected 36 images from the Open Affective Standardized Image Set (OASIS) database that uniformly spanned the complete range of the 1–7 beauty scale. Beauty ratings were collected for all 900 images in an independent study (Brielmann & Pelli, 2019). Here, we selected images based on beauty ratings because they reflect subjectively felt pleasure better than valence ratings. This is because valence ratings explicitly take the objective goodness or badness of the image content into account. For instance, the image of a sinking ship has low valence because its content is bad, while it can still be subjectively pleasant to look at the image because of its colors or composition. Here, we were interested in a purely subjective evaluation. Such beauty ratings are linearly related to subjectively felt pleasure (Brielmann & Pelli, 2017, 2019). To obtain images with a maximally broad and approximately uniform distribution of beauty ratings, the image set was divided into five beauty quintiles. To cover the extreme ends of the beauty range, the eight most beautiful images were selected from the first quintile and the eight least beautiful ones from the last quintile. A further eight images were selected from near the middle of each of the remaining three quintiles. A further four images, not presented in the main experiment, were selected from the middle quantile and presented on training trials.

Each image was assigned to one of two sets, A or B. For half of participants, Set A was presented on the left side and Set B on the right side (see Procedure section), and for the other half, Set B was presented left and Set A right. Four images from the middle quantile not presented in the main experiment were instead presented on training trials.

### Procedure

Participants viewed the images on a 27-in. iMac Retina LED display (58.2 cm × 36.4 cm, set to 1,600 px × 900 px) from a distance of approximately 1 m (so visual angles specified below are approximate). When white, the screen is 500 cd/m^2^. The room was normally lit. Participants were instructed to “rate how much pleasure you felt from this image (1–9).” It was emphasized that the content of the pictures was irrelevant, and that there were no right or wrong answers. Participants used the keys 1 through 9 on a standard keyboard to report pleasure from 1 (*no pleasure at all*) to 9 (*very intense pleasure*).

Fixation of a central fixation cross on a blank field is easy, and observers do this faithfully. The short stimulus duration of 200 ms ensured that no head or eye movement could occur before the stimulus ended. Participants were instructed to maintain central fixation whenever looking at the screen. The simultaneous brief presentation prevented a second glimpse. In the postcue condition, the random left/right position of the target made it impossible to reliably anticipate the target position.

**Fig. 1:**
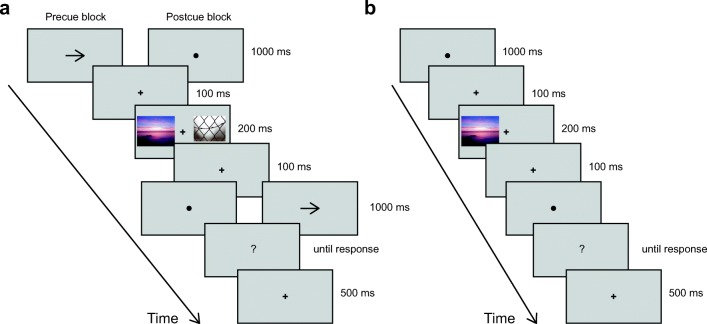
Time line for one example trial for the main experiment (**a**) and baseline ratings (**b**). **a** During the main experiment, participants were cued to rate either one of the images with an arrow pointing to the left (not shown), to the right (as shown), or to rate the combined pleasure of both images with a double-headed arrow (not shown)

Trial time lines for the different trial types are illustrated in Fig. 1. Images were presented for 200 ms in their original resolution (500 px × 400 px, about 10.4 deg × 9.3 deg). The short 200-ms presentation duration, well below the minimum reaction time for an eye movement (~250 ms), was chosen to avoid eye movements during the presentation. Thus, the two images were at symmetric eccentricities in the left and right periphery of the observer’s vision, avoiding greater attention to one image closer to the center of fixation. Subjectively felt pleasure from an image is reported reliably after presentation durations as short as 50 ms (e.g., Brielmann & Pelli, 2018; Forster, Leder, & Ansorge, 2016; Schwabe, Menzel, Mullin, Wagemans, & Redies, 2018).

Horizontally, the edge of each image nearest to fixation was 89 pixels (about 1.9 deg) from fixation (center of the mark), and its far edge was 51 px (about 1.1 deg) from the edge of the screen. Thus, when two images were shown, they were separated by a 2 × 249 px vertical gap (about 5.2 deg high), centered on fixation. A fixation cross of 20 px (about 0.4 deg) width and height was always present in the center of the screen, except while it was replaced by a cue or question mark. Cues were presented for 1,000 ms. Trials of all blocks had the same sequence and timing, differing only in the shape of the image cue (dot, left arrow, right arrow, or double arrow).

There are three kinds of blocks. In *precued* blocks, an arrow (left, right, or double) before image presentation indicates whether to rate the pleasure of the left or right image, or the combined pleasure of both images. In *postcued* blocks, the same arrows are presented after the images. In the final *baseline rating* block, only one image appears on the same side of the fixation cross as it did during precued and postcued blocks.

On each left-cued or right-cued trial, the cue points to the *target* image, and the other image is the *distractor*. In the final baseline rating block, only one image appeared in each trial. It was on the same side of the fixation cross as during precued and postcued blocks, but its position was not announced to participants.

Participants first practiced with six training trials, one for each possible image cue (left, right, or both), once precued and once postcued. As in the actual experiment, precued and postcued training trials were blocked and preceded by a note about whether the cues would appear before or after the images. After having the opportunity to ask the experimenter questions, observers completed four precued and four postcued blocks. In each block, each image was shown once as target, once as distractor, and once as part of a pair whose combined pleasure was rated (total *n* = 54 per block). At the beginning of each block, participants were told whether the cue would appear before or after the stimulus. Finally, participants completed the baseline rating block. They were then thanked, debriefed, and reimbursed. We assessed whether the ratings of a participant or for a particular image changed during the time course of the experiment to rule out sequence effects (see Supplementary Material Replication Study: Pleasure ratings are not influenced by sequence effects). By implication, this also rules out the possibility that baseline ratings at the end of the experiment were systematically corrupted by such sequence effects.

The precued condition encourages the participant to ignore the distractor and encode just the target pleasure. In contrast, the postcued condition demands that the participant encode two independent pleasure values, as either may be requested. Since the conditions differ only in when the cue appeared, any differences in pleasure rating between precued and postcued trials would indicate a limitation in the number of pleasure values that the participant can encode from a single glimpse.

### Analysis

Raw data, analyses files, and a more detailed description of all analyses are available at https://github.com/aenneb/tracking2pleasures. In the following, we will refer to an image’s rating from the final one-image block as its *single-pleasure*. We obtained the single-pleasure for every image presented during the cued trials. We model our data as a linear transformation of the weighted sum of target and distractor single-pleasures:

1 $$ \hat{P}=a+b\left(w{P}_1+\left(1-w\right){P}_2\right), $$

where $\hat{P}$ is reported pleasure, *w* is the target weight, 0.5 ≤ *w* ≤ 1, and *a* and *b* are constants. For one-pleasure trials, *P*_1_ represents target single-pleasure and *P*_2_ distractor single-pleasure. For combined-pleasure trials, *P*_1_ represents the left image’s single-pleasure and *P*_2_ the right image’s single-pleasure.

All models discussed in the introduction correspond to special cases of Equation 1. The main models of interest for one-pleasure trials are the faithful, compulsory averaging, and partial compulsory averaging models. These models assume no linear transformation (i.e., *a* = 0 and *b =* 1). Crucially, the compulsory averaging model has a weight *w =* 0.5, whereas a faithful has *w* = 1. Values for *w* fall in between for the partial compulsory averaging model.

The main models for combined-pleasure trials are the faithful, compressive, and expansive models. All three models assume *w =* 0.5. The compressive model predicts a slope of 0 < *b* < 1, and an intercept *a* > 0. The expansive model predicts a slope of *b* > 1 and an intercept of *a* < 0. In contrast, the faithful model predicts an intercept of *a* = 0 and a slope of *b =* 1. Note that averaging the image pleasures corresponds to *b* = 1, whereas summing would correspond to *b* = 2. Illustrations of all model predictions appear in the Results section.

All models derived from Equation 1 aim to explain the relation between *mean* pleasure ratings on single-pleasure, one-pleasure, and combined-pleasure trials. They do not refer to variance. We explore the modeling of rating variances in the Supplementary Material: Accounting for rating variances.

As a measure for reliability, we calculated Cronbach’s alpha for precued and postcued trials. Cronbach’s alpha measures intercorrelations among items (here, image ratings) to gauge internal consistency. We chose this analysis because it has previously been applied by Habermann, Brady, and Alvarez (2015) who also compared perception of single items with the perception of their average. Repeating their analysis strategy makes our results directly comparable. That is, for one-pleasure trials, we calculated the absolute deviation of each trial’s pleasure rating from the target single-pleasure. For combined-pleasure trials we calculated absolute deviation of pleasure reports from the mean across single-pleasures of the two presented images. We then averaged absolute errors per participant. Data from one participant was excluded from the reliability analyses due to missing values for single-pleasure (due to hitting an invalid key).

Intraclass correlation coefficient (ICC)—here, the degree of absolute agreement among measurements—was calculated using the MATLAB script provided by Arash Salarian (https://www.mathworks.com/—matlabcentral/fileexchange/22099-intraclass-correlation-coefficient-icc).

Leave-one-out cross-validation (LOOCV) analyses were conducted for each individual participant separately. We used several models based on Equation 1. The models were fit to all but the one left-out trial. The models were distinguished by their different permissible ranges of the model parameters *a*, *b*, and *w* as described above. To fit these models, we used the built-in MATLAB function fmincon(). The cost function for the minimization problem was the root mean square error (RMSE) between model predictions and observed values for ratings on individual trials. For each model and each observer, we calculated the average RMSE between model predictions and observed rating in the left-out test trials. Last, we calculate the mean RMSE per model across participants as well as the standard error of this mean.

## Results

Results of the replication follow. The complete results of the original pilot study are in the Supplementary Results Pilot Study. Both studies used the same methods.

Based on the findings of our pilot study, we preregistered five hypotheses, listed below. All five are confirmed by our results.

1. Cronbach’s alpha for one-image trials is greater than or equal to that for both-image trials.
2. A high correlation, close to the maximal obtainable value, between errors of precued and postcued one-image trials, and a high correlation, close to the maximal obtainable value, between errors of precued and postcued both-image trials.
3. No correlation between errors of one-image and both-image trials.
4. The faithful (originally “accurate observer”) model (which has no degrees of freedom) fits the data of one-image trials better than linear models (which have at least one degree of freedom; i.e., the average RMSE of the faithful model is lower than or equal to that of linear models).
5. The faithful model fits the mean ratings of both-image trials best i.e., yields an average RMSE less than or equal to that of compressive or expansive models. Yet, the variance of the observer’s report is similar whether reporting pleasure of one image or the average pleasure of two images.

### Single-pleasure and reliability

To test whether our stimulus selection was effective for the participants in the current experiment, we correlated pleasure ratings in the final single-image block with the standardized valence (Kurdi, Lozano, & Banaji, 2016) and beauty (Brielmann & Pelli, 2019) ratings. We found very high positive correlations for both measures: mean *r =* .81 and *r =* .80 for beauty and valence, respectively, with minimum *r* = .07 and *r* = .06, and maximum *r* = 0.90 and *r* = .92. Interrater reliability for single-pleasure as measured via the intraclass correlation coefficient was moderate, ICC = 0.67. This indicates that, even though single-pleasure ratings were closely related to standardized beauty and valence ratings, there was still considerable variation between participants’ subjective pleasure ratings. Therefore, all our analyses use within-participant single-pleasure ratings and fit our models for each participant individually.

Cronbach’s alpha is a widely used measure of internal consistency that increases as the intercorrelations among test items increases. Here, we computed alpha for the mean absolute error per item (see Method section). As predicted by Hypothesis 1, alpha for all kinds of trial was high, and those for one-image trials (precued α = 0.92; postcued α = 0.89) were not smaller than the ones for both-image trials (precued α = 0.85; postcued α = 0.81). The fact that participants were not more reliable when rating the average across two images suggests either that the variance of simultaneous pleasures is highly correlated or, more likely, that the variance in ratings does not arise in sampling the pleasure of each image (if so, averaging would reduce variance). Instead, this finding suggests that the variance arises later, either during the computation of average pleasure or in the response stage.

We also explored whether the repeated presentation of each image influenced pleasure ratings. Overall, habituation or mere exposure effects were minimal (see Supplementary Material Replication Study: Pleasure ratings are not influenced by sequence effects). Therefore, we did not include sequence effects in our models.

### Correlations between tasks

The reliabilities reported above place an upper bound on the correlation between errors in the two kinds of trial (Nunnally, 1970):

2 $$ {r}_{X,Y}\ge \sqrt{\alpha_X{\alpha}_Y}, $$

where *X* and *Y* are two random variables (in our case, the errors in two kinds of trial) and *α* is Cronbach’s alpha.

Inserting the values for Cronbach’s alpha reported above into Equation 2, we obtain estimates of the maximally achievable correlation between different trial types. These maximum correlations are 0.91 between precued and postcued one-pleasure trial performances and 0.83 between precued and postcued combined-pleasure trials, 0.89 between one-pleasure and combined-pleasure postcued trials, and 0.85 between one-pleasure and combined-pleasure precued trials. As predicted, we find that correlations within one-pleasure and combined-pleasure trials are at maximum given the limited reliability, *r* = .93 and *r* = .92, respectively. We predicted zero, but found moderate correlation between errors of one-pleasure and combined-pleasure in precued and postcued trials, *r* = .52 precued and *r* = .45 postcued. However, these correlations are small compared with the correlations between precued and postcued trials (*r* = .93 and *r* = .92, respectively). The absence of a correlation between error rates in one-image and both-image trials would suggest a different source of noise or error involved in judging the pleasure of one image versus the average across two. As mentioned above, it may be that additional noise arises during the computing or reporting of the average.

### In one glimpse, people can tell the pleasure of each of two images

We fit three models to each participant’s data: (1) the faithful model, (2) the compulsory averaging model, and (3) a flexible model that allows the weights given to target and distractor to take any values that add up to 1. To avoid overfitting, we performed leave-one-out cross validation (LOOCV) with RMSE as the statistic to assess the goodness of fit of the three models.

**Fig. 2:**
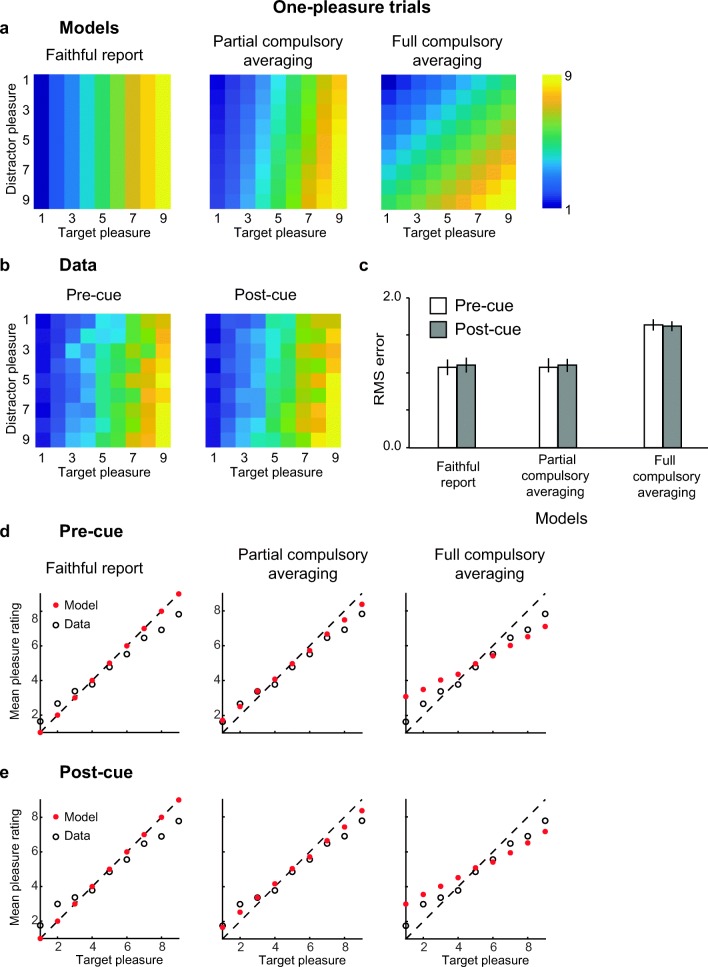
Model predictions (**a**), data (**b**), and RMSE (**c**) for one-pleasure trials. **a–b** Heat maps show the predicted (**a**) and average (**b**) pleasure ratings for each possible combination of target and distractor pleasures. Each cell represents the average pleasure rating (**a**) or predicted rating (**b**) per target and distractor pleasure combination. Cooler colors indicate lower average ratings, warmer colors indicate higher ones. Note that the averaged data still take interindividual differences into account since target-pleasure and single-pleasure are assigned to each image according to the individual observer’s own ratings. Predictions for the partial averaging model are displayed for the average value of the weight parameter that was the best fit (setting the weight variable of equation (1) to *w =* 0.8). **c** Root mean square error averaged across observers for precued (white) and postcued (gray) trials. Error bars represent ±1 *SEM*. **e–d** Average predicted (filled red circles) and observed ratings (open black circles) for precued (**d**) and postcued trials (**e**). Each dot represents averaged values across the same target rating value

As illustrated in Fig. 2 the pattern of pleasure ratings was highly similar to the predictions of the faithful model. This graphic impression is confirmed by the average RMSEs of each model. The pattern of results did not differ between precued and postcued trials, suggesting that selective tracking, which is only possible with precuing, is not necessary for reporting the pleasure of a single image in a set of two. As predicted, even though the more flexible partial compulsory averaging model has one additional free parameter, it did not fit the data better than the faithful model, mean RMSE = 1.10 and 1.09 for the both models in precued and postcued trials, respectively. This shows that observers can ignore the pleasure of an irrelevant distractor. The results were highly consistent across participants (see Supplementary Replication Study: Consistency of results across participants). We further explored whether distractors might influence pleasure reports differentially depending on baseline target pleasure, but again we found that these alternative models do not outperform the faithful model (see Supplementary Replication Study: Exploratory analyses).

### People can average pleasure across two images

Analyses for trials in which observers were cued to report the combined pleasure of the pair of images followed the same logic as for one-pleasure trials. Here we tested the performance of three models: (1) faithful, (2) compressive, and (3) expansive. All models are variations of Equation 1. All models assume *w =* 0.5. The compressive model supposes a slope of 0 < *b* < 1, and an intercept *a* > 0. The expansive model supposes a slope of *b* > 1 and an intercept of *a* < 0. In contrast, the faithful model supposes an intercept of *a* = 0 and a slope of *b =* 1.

**Fig. 3:**
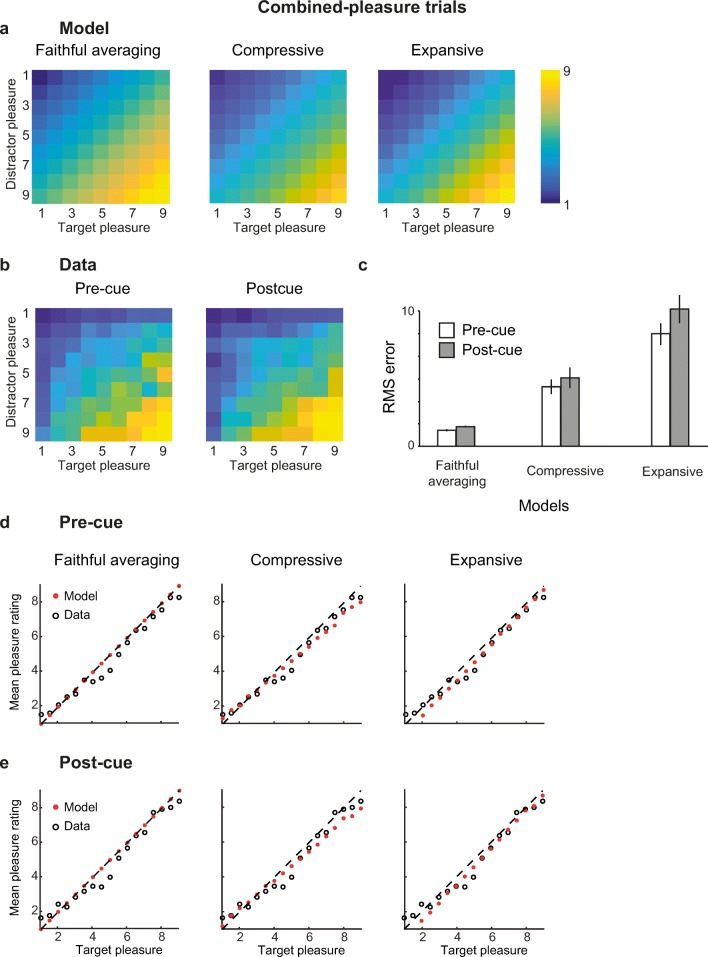
Model predictions (**a**), data (**b**), and RMSE (**c**) for combined-pleasure trials. **a–b** Heat maps show the predicted (**a**) and average (**b**) pleasure ratings for each possible combination of target and distractor pleasures. Note that the averaged data still take interindividual differences into account since target-pleasure and single-pleasure are assigned to each image according to the individual observer’s own ratings. Predictions for the compressive and expansive model are displayed for the average value of the best fitting parameters (*a =* 0.39 and *b* = 0.85 for the compressive model; *a =* −0.70 and *b* = 1.05 for the expansive model). **c** Root mean square error averaged across observers for precued (white) and postcued (gray) trials. Error bars represent ±1 *SEM*. **e–d** Average predicted (filled red circles) and observed ratings (open black circles) for precued (**d**) and postcued trials (**e**). Each dot represents averaged values across the same target rating value

The model predictions are illustrated in Fig. 3. As predicted, the faithful model fits better than the more complex models in both precued and postcued trials. RMSEs for the faithful model of averaging were both below 1.4, while all other exceeded 4.7. Again, results were highly consistent across participants (see Supplementary Replication Study: Consistency of results across participants).

Recall that in the combined-pleasure trials, the observers were merely asked to report the “combined” pleasure, with no mention of “averaging.” In principle the combined pleasure they reported might have been the sum of the individual pleasures. The success of the averaging model (*b* = 1) rejects the summing hypothesis (*b* = 2), showing that observers reported the average pleasure.

### Rating variance is stable for one-pleasure and combined-pleasure ratings

Apart from looking at participants’ mean ratings, we also looked at the variance of their ratings (see https://github.com/aenneb/tracking2pleasures for standard deviations per participant, target pleasure, and trial type). Average standard deviations for one-pleasure and combined-pleasure ratings were nearly identical for both precued (1.41 vs. 1.33 for one-pleasure and combined-pleasure ratings, respectively) and postcued trials (1.40 vs. 1.35), both *p*s ≥ .899, two-tailed *t* test on averages per participant. This result stands in contrast to the standard deviation expected if participants were averaging across two sampled pleasures and report that value without any further added variance. In that case, we would expect a variance reduction by a factor of the square root of two.

The model described in this article were developed to account for the relation between mean pleasure ratings. We report the results of exploratory analyses of a model that may account for the pattern of variances in the Supplementary: Accounting for rating variances.

## Discussion

All the main results of the original study with 13 observers (Supplementary Pilot Study) were replicated with 25 observers (Results).

We find that people can report their felt pleasure from each of two simultaneous brief images as faithfully as when only one image is presented. People can also report the average pleasure across two simultaneously presented images (though without the reduction in variance expected from averaging). This averaging is optional, not compulsory. They can tell the pleasure of each image in a brief presentation whether or not they know in advance which pleasure to report, implying that participants can track two pleasures from one glimpse.

### Unbiased reporting of each pleasure and the average of both when viewing two images

People are biased toward the average of an array when reporting properties (e.g., size or color) of a single item (Brady & Alvarez, 2011; Corbett, 2017; Haberman & Whitney, 2009; Maule et al., 2014; Parkes et al., 2001). So far, it has not been assessed whether people have a similar bias when reporting their subjective feelings about a stimulus. Here, we show that people can report the pleasure of an image presented simultaneously with another. This was true for precued trials in which participants could selectively track just the target image, and for postcued trials, in which participants needed to track both images from one glimpse. This shows that observers can encode and track the pleasure of two images that were presented simultaneously in one glimpse (200 ms). Thus, whereas the report of objective object features, such as orientation (e.g., Parkes, et al., 2001), size (de Fockert & Marchant, 2008), position (Alvarez & Oliva, 2008), motion (e.g., Watamaniuk, 1993), and number (e.g., Burr & Ross, 2008), of one object among many is biased, the report of the subjective pleasure is not, at least not when only one other image is shown. Alas, differences in methods make it hard to compare our findings on variance of subjective pleasure ratings with earlier studies of variance of estimates of objective qualities. To make it easier to compare these variances, the response type (rating, adjustment, two-alternative forced choice, etc.) and number of stimuli should be the same for pleasure and the other feature. We hope to address this in a future study.

Our stimuli included the most beautiful and most pleasant as well as the least beautiful and most unpleasant OASIS images (Kurdi et al., 2016). Thus, our stimulus selection was suitable to detect the effects predicted by partial averaging. Still, we did not find evidence that the partial-averaging model performed better than the simpler faithful model.

Participants could also report the average pleasure from two images. When asked to rate the combined pleasure of two images, observers’ ratings were best predicted by the average of the two images’ isolated pleasure ratings. However, the variance of these ratings is about as high as when they rate the pleasure of one image, suggesting that the response variance arises in the shared combining or reporting, not in the presumed independent variance of estimating each pleasure. This is in line with previous findings for reports of average size (Allik et al., 2013) and other studies that find that set size does not influence the accuracy of reports of the average of low-level visual features (e.g., Ariely, 2001; Chong & Treisman, 2005).

### Source of variance

We find that the variance of the observer’s report is similar whether reporting the pleasure of one image or the average pleasure of two images. This rejects the hypothesis that the variance of the observer’s report of average pleasure is simply the average of two stochastically independent pleasures, because the averaging would halve the variance. Instead, the conservation of variance across set size (1 or 2) indicates that either the two pleasure variances are highly correlated, or, more likely, that the report variance arises in the common reporting process rather than in the separate pleasures.

### Implications

As far as we know, this is the first study of people’s ability to report subjective pleasure experienced from several images in a single glimpse. Regardless of precuing or postcuing, we found consistent mean rated pleasure of one image alone, one of two images, and the combined pleasure of both.

On a pragmatic note, finding that observers can tell two pleasures as well as they tell one, promises a coherent account across experiments with single and multiple stimuli, and helps to validate extrapolation from laboratory studies with one stimulus to a world with many (e.g., in museums; Brieber, Nadal, Leder, & Rosenberg, 2014; Van Paasschen, Bacci, & Melcher, 2015), as they suggest that participants can ignore surrounding stimuli while judging the one stimulus of interest.

We find that the experience of pleasure is not univariate (just one global pleasure). Instead, people can individuate felt pleasures for each of at least two objects. The assumption that pleasure is univariate is often made, for instance, when applying a physiological measure, such as facial electromyography, as a proxy for the pleasure felt from a stimulus. As these measures yield only one number at a time, they cannot disentangle parallel responses to simultaneously experienced objects. We show that people can access any one of several sources of pleasures from a glimpse. It remains to be determined which aspects of the parallel pleasures of multiple stimuli can be captured by physiological methods.

Another promising avenue for future research is to extend our unimodal vision findings to multimodal stimuli. Can people process the pleasure of, for example, a song and an image in parallel? We think this is plausible for three reasons. Firstly, it is generally supposed that presenting two stimuli through different senses enhances independent processing. Secondly, the efficiency of cue combination mostly depends on whether the cues relate to the same object, not to the same modality (Dubois, Poeppel, & Pelli, 2013). Thirdly, pleasures, or rewards, from different modalities are processed partially within the same areas of the brain (e.g., Brown et al., 2001).

### Conclusion

All the main results of the original study with 13 observers were replicated with 25 observers. In sum, our results show that people shown two brief images simultaneously can give unbiased reports of their felt pleasure from each and the average of both. Not only can people voluntarily track one of two images and give an unbiased report of the pleasure felt from it in the presence of a distractor. They can also retain the pleasures of both and report either one.

Furthermore, we find that the variance of the observer’s report is similar whether reporting the pleasure of one image, presented in isolation or as one of a pair, or reporting the average pleasure of two images. The undiminished variance for reports of the average pleasure of two images indicates either that the pleasure variances are highly correlated, or, more likely, that the variance arises in the common reporting process.

## Electronic supplementary material

ESM 1 (DOCX 847 kb)
