# Supplementary material for: Tracking two pleasures
Source: Psychon Bull Rev. 2020 Jan 2;27(2):330–40. doi: 10.3758/s13423-019-01695-6 (PMC7093342; doi:10.3758/s13423-019-01695-6)
Supplement: Supplementary file 1 — (DOCX 847 kb) [file 13423_2019_1695_MOESM1_ESM.docx]

Tracking two pleasures

Aenne A. Brielmann & Denis G. Pelli

**SUPPLEMENTARY RESULTS PILOT STUDY**

The procedures of the original and replication were identical.

**Pilot study single-pleasure and reliability**

To test whether our stimulus selection was effective for the participants in the current experiment, we correlated pleasure ratings in the final single-image block with the standardized valence (Kurdi, Lozano, & Banaji, 2016) and beauty (Brielmann & Pelli, under review) ratings. We found very high positive correlations for both measures: mean *r =*0.84 and *r =*0.81 for beauty and valence respectively, with minimum *r* = 0.63 and *r* = 0.56, and maximum *r* = 0.92. Inter-rater reliability for single-pleasure as measured via the intraclass correlation coefficient was moderate, ICC = 0.62. This indicates that, even though single-pleasure ratings were closely related to standardized beauty and valence ratings, there was still considerable variation between participants’ subjective pleasure ratings. Therefore, all our analyses use within-participant single-pleasure ratings and fit our models for each participant individually.

Cronbach’s alpha is a widely used measure of internal consistency that increases as the intercorrelations among test items increases. Here, we computed alpha for the mean absolute error per item (see **Methods**). Alpha for all kinds of trials was high (one-pleasure trials pre-cued α = 0.87; post-cued α = 0.90; combined-pleasure trials pre-cued α = 0.73; post-cued α = 0.80). The fact that participants were not more reliable when rating the average across two images suggests either that the variance of simultaneous pleasures is highly correlated, or, more likely, that the variance in ratings does not arise in sampling the pleasure of each image (if so, averaging would reduce variance). Instead, this finding suggests that the variance arises later, either during the computation of average pleasure or in the response stage.

We also explored whether the repeated presentation of each image influenced pleasure ratings. Overall, habituation or mere exposure effects were minimal (see below). Therefore, we did not include sequence effects in our models.

**Pilot study correlations between tasks**

The reliabilities reported above place an upper bound on the correlation between errors in the two kinds of trial (Nunnally, 1970):

$r_{X,Y}\geq\sqrt{\alpha_{X}\alpha_{Y}}$ (2)

where *X* and *Y* are two random variables (in our case the errors in two kinds of trial) and *α* is Cronbach’s alpha.

Inserting the values for Cronbach’s alpha reported above into Eq.2, we obtain estimates of the maximally achievable correlation between different trial types. These maximum correlations are 0.88 between pre- and post-cued one-pleasure trial performances and 0.76 between pre- and post-cued combined-pleasure trials, 0.84 between one- and combined-pleasure post-cued trials, and 0.80 between one- and combined-pleasure pre-cued trials. We find that correlations within one- and combined-pleasure trials are close to maximum given the limited reliability, *r* = 0.81 and *r* = 0.65 respectively. In contrast, there was no correlation between errors of one- and combined-pleasure in pre-cued trials, *r* = ­ 0.05, and only a weak one for post-cued ratings, *r* = 0.22. Thus, there is a different source of noise or error involved in judging the pleasure of one image versus the average across two. As mentioned above, it may be that additional noise arises during the computing or reporting of average pleasure.

**Pilot study: In one glimpse, people can tell the pleasure of each of two images**

We fit three models to each participant’s data: 1) the faithful model, 2) the compulsory averaging model, and 3) a flexible model that allows the weights given to target and distractor to take any values that add up to 1. To avoid overfitting, we performed leave-one-out cross validation (LOOCV) with RMS error as the statistic to assess the goodness of fit of the three models.

As illustrated in **Figure S1**, the pattern of pleasure ratings was highly similar to the predictions of the faithful model. This graphic impression is confirmed by the average RMS errors of each model. The pattern of results did not differ between pre- and post-cued trials, suggesting that selective tracking, which is only possible with pre-cueing, is not necessary for reporting the pleasure of a single image in a set of two. Even though the more flexible partial compulsory averaging model has one additional free parameter, it did not outperform the faithful model, mean RMS error = 0.93 and 0.90 for the faithful model in pre- and post-cued trials respectively versus RMS error = 0.95 for the partial averaging model in both trial types. This shows that observers can ignore the pleasure of an irrelevant distractor. The results were highly consistent across participants (see below). We further explored whether distractors might influence pleasure reports differentially depending on baseline target pleasure but again found that these alternative models do not outperform the faithful model of reporting (see below).


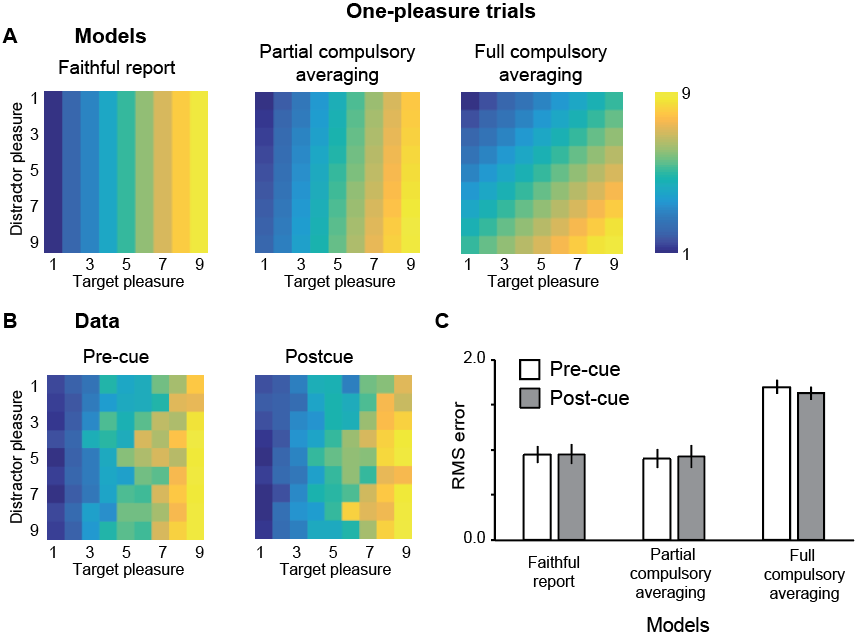


*Figure S1.* Model predictions (A), data (B), and RMS error (C) for one-pleasure trials. A-B) Heat maps show the predicted (A) and average pleasure ratings (B) for each possible combination of target and distractor pleasures. Note that the averaged data still takes inter-individual differences into account since target- and single-pleasure are assigned to each image according to the individual observer’s own ratings. Predictions for the partial averaging model are displayed for the average value of the weight parameter that was the best fit (*w =*0.87). C) Root mean square error (RMS error) averaged across observers for pre-cued (white) and post-cued (gray) trials. Error bars represent ± 1 SEM.

**Pilot study: People can average pleasure across two images**

Analyses for trials in which observers were cued to report the average pleasure of the pair of images followed the same logic as for one-pleasure trials. Here we tested the performance of three models: 1) faithful, 2) compressive, and 3) expansive. All models are variations of Eq 1. All models assume *w =*0.5. The compressive model supposes a slope of 0 < *b* < 1, and an intercept *a* > 0. The expansive model supposes a slope of *b* > 1 and an intercept of *a* < 0. In contrast, the faithful model supposes an intercept of *a* = 0 and a slope of *b =*1.

The model predictions are illustrated in **Figure S2**. The faithful model outperforms the more complex models in both pre- and post-cued trials. RMS errors for the faithful model of averaging were both below 1.4 while all other exceeded 2.9. Again, results were highly consistent across participants (see below).


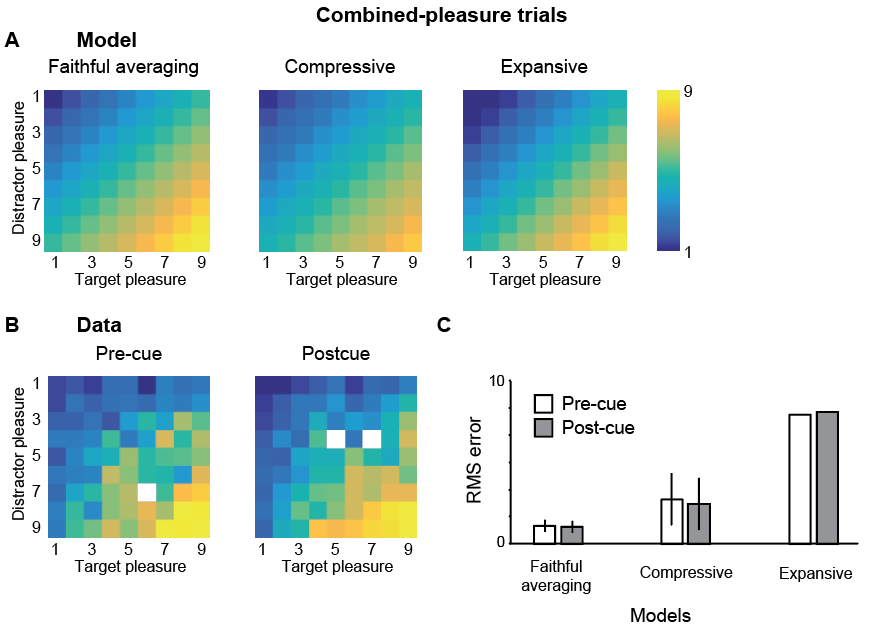


*Figure S2.* Model predictions (A), data (B), and RMS error (C) for combined-pleasure trials. A-B) Heat maps show the predicted (A) and average pleasure ratings (B) for each possible combination of target and distractor pleasures. Each cell represents the average pleasure rating (A) or predicted rating (B) per target and distractor pleasure combination. Cooler colors indicate lower average ratings, warmer colors higher ones. White cells indicate cells with missing data due to the absence of the target and distractor pleasure combination in the data. Note that the averaged data still takes inter-individual differences into account since target- and single-pleasure are assigned to each image according to the individual observer’s own ratings. Predictions for the compressive and expansive model are displayed for the average value of the best fitting parameters (*a =*0.30 and *b* = 0.85 for the compressive model; *a =*–0.88 and *b* = 1.09 for the expansive model). C) Root mean square error (RMS error) averaged across observers for pre-cued (white) and post-cued (gray) trials. Error bars represent ± 1 SEM.

**Pilot study: Pleasure ratings are not influenced by sequence effects**

We assessed whether the ratings of a participant or for a particular image changed during the time course of the experiment to rule out sequence effects and therewith also the possibility that baseline ratings at the end of the experiment were systematically corrupted by such sequence effects.


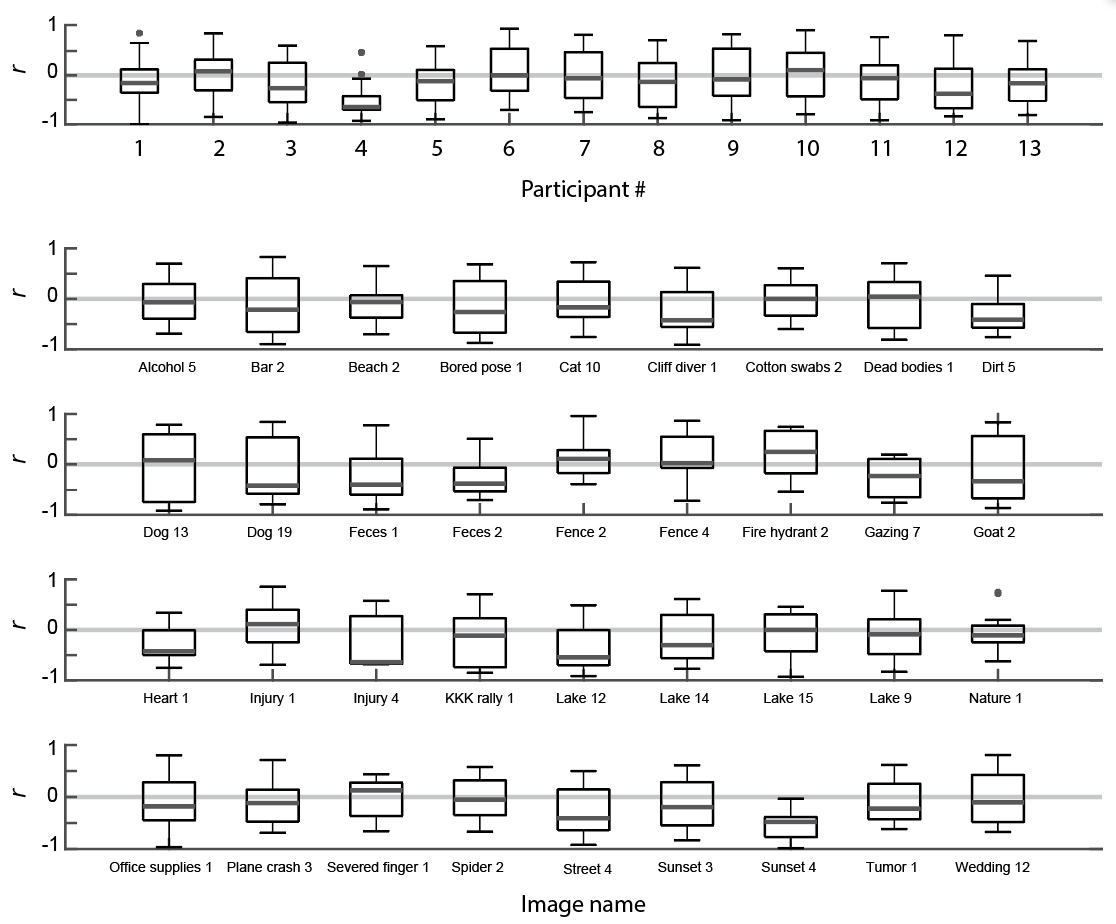


*Figure S3.* Boxplots of correlation coefficients for the relationship between pleasure and image repetition across images per participant (top) and across participants per image (remaining rows). Sequence effects were only evident for participant #4 and the image “sunset 4”; both times, pleasure ratings decreased over the time course of the experiment.

**Pilot study: Consistency of results across participants**

**Figure S4** shows the comparison between all tested models’ RMSEs for each individual participant. The pattern of results is highly consistent for all 13 observers: The faithful models’ errors (white bars) are never meaningfully higher than those of the other models with more free parameters. The compulsory averaging and the extreme bias model are always the worst fit for the data.


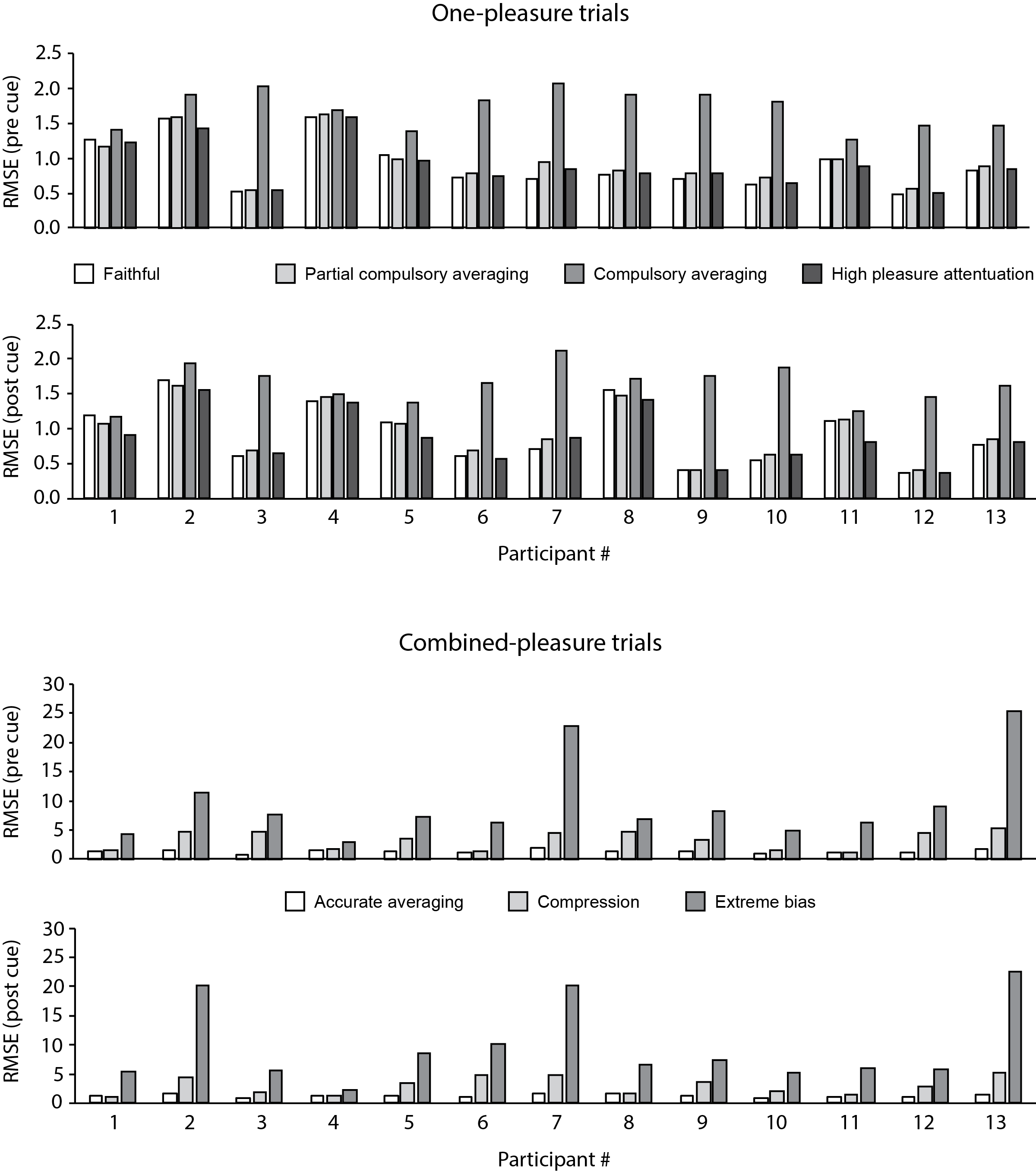


*Figure S4.* Average root mean square error (RMSE) per participant and candidate model based on leave one out cross validation.

**Pilot study: Exploratory analyses**

The modeling procedures described above show that of the three conjectures, the faithful model describes participants’ behavior best. However, we also wanted to explore whether there could be distractor influences that follow a different pattern. To do so, we conducted a linear regression predicting average pleasure per target and distractor pleasure. As for previous analyses, target and distractor pleasure were assigned based on each individual participant’s single-image ratings. The linear model was fit with the aov function (R version 3.5.3) with both target and distractor pleasure converted to a factor to enable the detection of non-linear interactions. The resulting model (Eq. S1) predicted pleasure ratings well, *F*(3, 525) = 600.6, *p* < 0.001, *R*_adj_^2^ = 0.77.

$\hat{P}=0.91+0.65P_{1}-0.04P_{2}+0.03P_{1}P_{2}$ (S1)

As expected, target pleasure was the main predictor of pleasure ratings, β = 0.65, *p* < 0.001. Distractor pleasure had no main effect, as would be predicted by a compulsory averaging model, β = –0.04, *p* = 0.302. Instead, target and distractor pleasure interacted, β = 0.03, *p* < 0.001. To better understand the interaction, we plotted average pleasure ratings as a function of distractor pleasure separately per target pleasure (see **Figure S5A**). The pattern visible there indicates that low-pleasure distractors decreased pleasure ratings for high- but not low-pleasure targets.


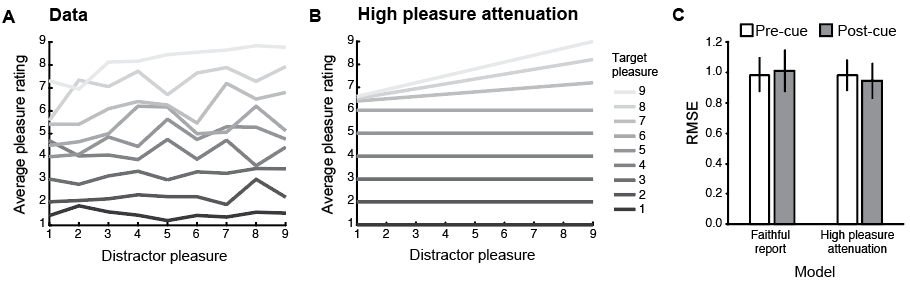


*Figure S5.* Illustration of exploratory analyses of a high-pleasure attenuation model. A) Average pleasure ratings across observers per distractor pleasure calculated per target pleasure. B) Predictions of the high pleasure attenuation model (Eq. S2) with *P*_beau_ = 6.3, the average of the best-fitting value. A) and B) The darker the lines, the lower the target pleasure for the averages. C) Root mean square error (RMS error) averaged across observers for pre-cued (white) and post-cued (gray) trials. Error bars represent ±SEM.

We therefore decided to test an additional model that allows for distractor-dependent attenuation of higher pleasures only. This model mimics the effect of a distracting task on high pleasure and beauty (Brielmann & Pelli, 2017). It assumes that reported pleasure is only affected by distractor pleasure if the target pleasure exceeds a threshold *P*_beau_. The weight of the distractor pleasure then increases with increasing target pleasure. This high-pleasure attenuation model is defined by:

$\hat{P}=\left\{ \begin{aligned} P_{1} \mathrm{if}P_{1}<P_{\mathrm{beau}} \\ P_{\mathrm{beau}}+\frac{P_{2}}{P_{1}}\left( P_{1}-P_{\mathrm{beau}} \right) \mathrm{if} P_{1}\geq P_{\mathrm{beau}} \end{aligned} \right.$ (S2)

where $\hat{P}$ is reported pleasure. For one-pleasure trials, *P*_1_ represents target single-pleasure and *P*_2_ distractor single-pleasure. For combined-pleasures trials, *P*_1_ represents the left image’s single-pleasure and *P*_2_ the right image’s single-pleasure.

As in our main analyses, we used LOOCV to fit the high-pleasure attenuation model. For these analyses, data was fit and tested for each participant separately. On average, across participants, the best fitting model had a threshold pleasure *P*_beau_ = 6.60 ± 0.49 (mean ± SE), for pre-cued trials and *P*_beau_ = 6.01 ± 0.57, for post-cued trials. We again found that the average RMS error for this more sophisticated model was no lower than for the faithful model (see **Figure S5B**).

**Pilot study: Accounting for rating variances**

In contrast to the predictions of our faithful model, SDs for one- and combined-pleasure ratings were nearly identical. Furthermore, when we looked at the variance of ratings depending on the expected rating, we found that combined-pleasure rating variances were not constant across ratings but followed an inverted u-shaped pattern (see black data points in **Figure S6**).

Searching for an explanation for this pattern, we came up with a modified faithful model that can reproduce this pattern (see orange data points in Figure **Figure S6**). Like the model presented in the main paper, the modified model assumes that participants report the single-pleasure of the target image in one-pleasure trials and the average pleasure of both presented images in combined-pleasure trials. Crucially, the model also contains a lapse-rate component, i.e., it assumes that on a certain proportion of trials, participants experience a lapse of attention and therefore respond with a random number (on the 1-9 scale). Pleasure responses for such lapse trials are sampled randomly from a uniform distribution.

The lapse rate model that fit our data best assumes a lapse rate that is the product of 4% and the participant’s average pleasure rating *SD* across target ratings. Lapse rates for participants ranged from 3.98% to 8.04%. Figure S6 shows a good fit between lapse rate model predictions and observed SDs, especially for the inverted-u shaped pattern in combined-pleasure trials.


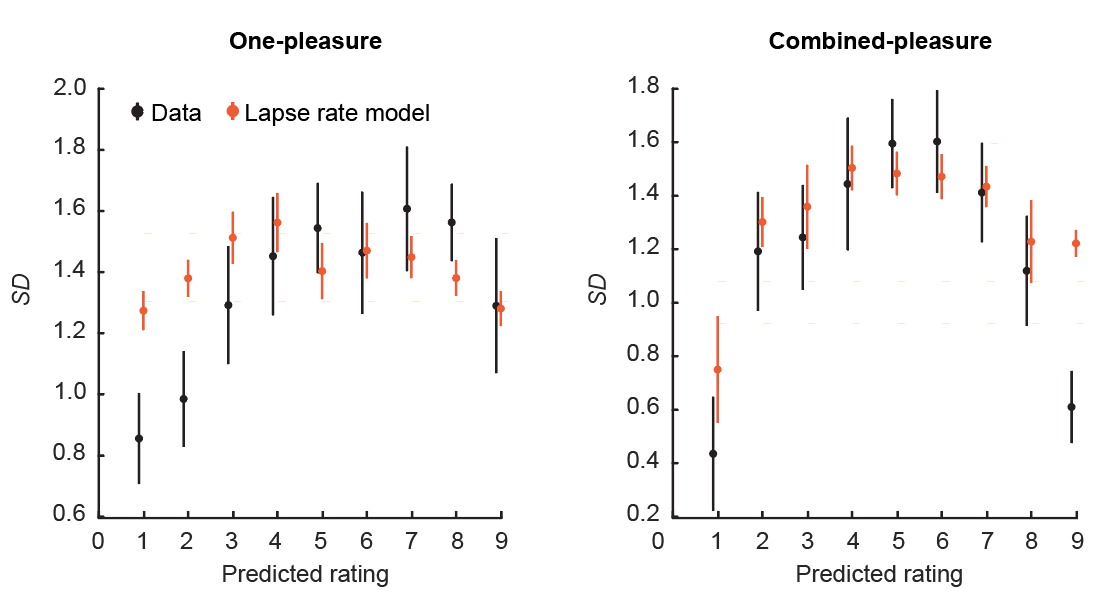


*Figure S6.* Average *SD*s per predicted rating for one- (left) and combined-pleasure trials (right). Predicted ratings refer to the single-image target pleasure for one-pleasure trials and to the average of both images’ single-pleasures for combined-pleasure trials. Data is shown in black, predictions of the lapse rate model in orange. Predictions of the lapse rate model were based on the average of 100 iterations of simulations run separately for each participant using the trial sequence each participant encountered. Error bars represent ±SEM.

**SUPPLEMENTARY RESULTS REPLICATION STUDY**

**Pleasure ratings are not influenced by sequence effects**


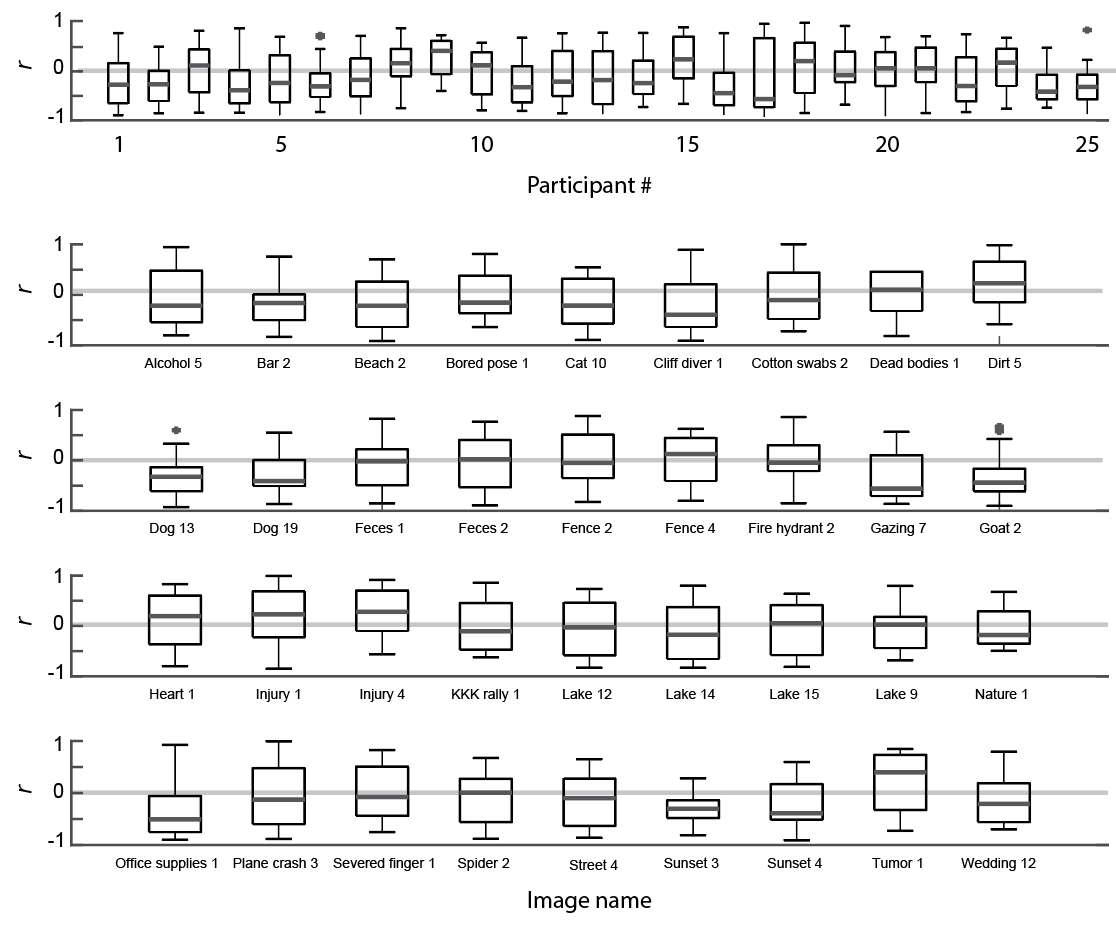


*Figure S7.* Boxplots of correlation coefficients for the relationship between pleasure and image repetition across images per participant (top) and across participants per image (remaining rows). Sequence effects were not evident for any participant or image.

**Consistency of results across participants**

**Figure S8** shows the comparison between all tested models’ RMSEs for each individual participant. The pattern of results is highly consistent for all 25 observers: The faithful models’ errors (white bars) are never meaningfully higher than those of the other models with more free parameters. The compulsory averaging and the extreme bias model are always the worst fit for the data.

**
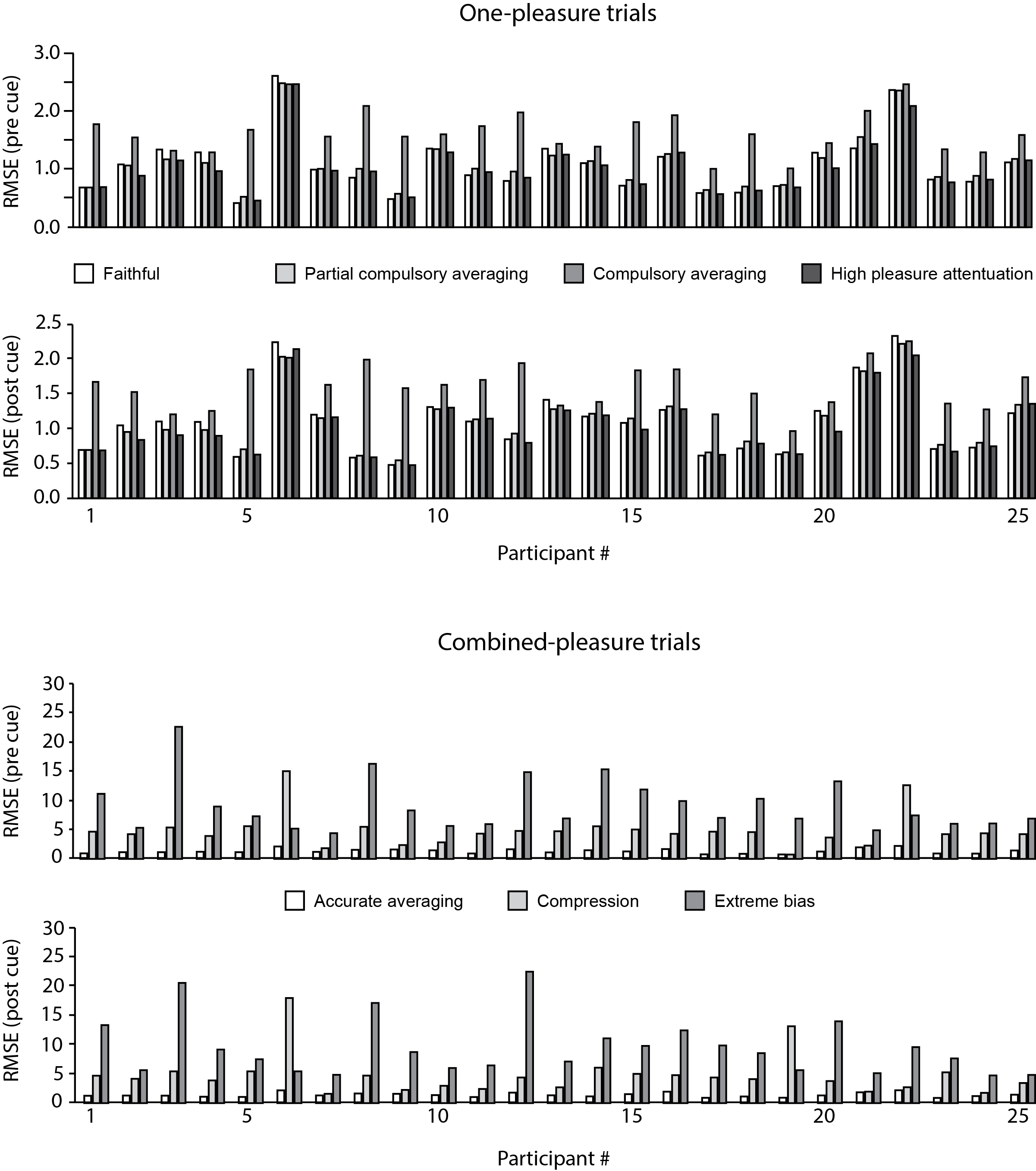
**

*Figure S8.* Average root mean square error (RMSE) per participant and candidate model based on leave one out cross validation.

**Exploratory analyses**

We repeated our exploratory analyses of a high pleasure attenuation model with the replication data (see **Figure S9**). We again found that the average RMS error for this more sophisticated model was no lower than for the faithful model (see **Figure S9B**).


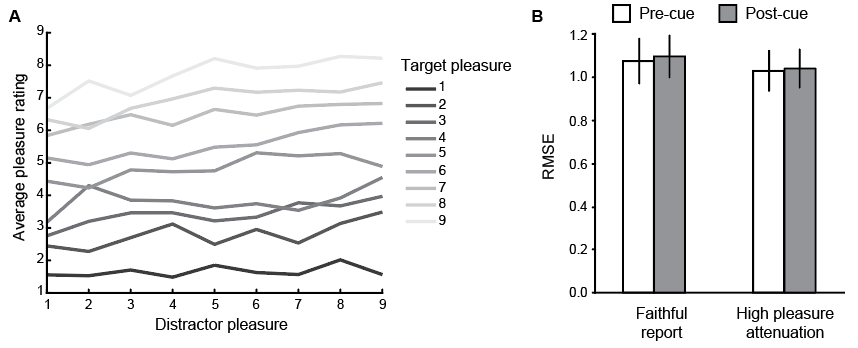


*Figure S9.* Illustration of exploratory analyses of a high-pleasure attenuation model. A) Average pleasure ratings across observers per distractor pleasure calculated per target pleasure. The darker the lines, the lower the target pleasure for the averages. B) Root mean square error (RMS error) averaged across observers for pre-cued (white) and post-cued (gray) trials. Error bars represent ±SEM.

**Accounting for rating variances**

As in the pilot data, SDs for one- and combined-pleasure ratings were nearly identical in the replication data. Again, we found that combined-pleasure rating variances were not constant across ratings but followed an inverted u-shaped pattern (see black data points in **Figure S10**).

A full description of the lapse rate model that can account for this pattern is given above (**Pilot study: Accounting for rating variances**). Here, lapse rates for participants ranged from 3.53% to 9.81%.


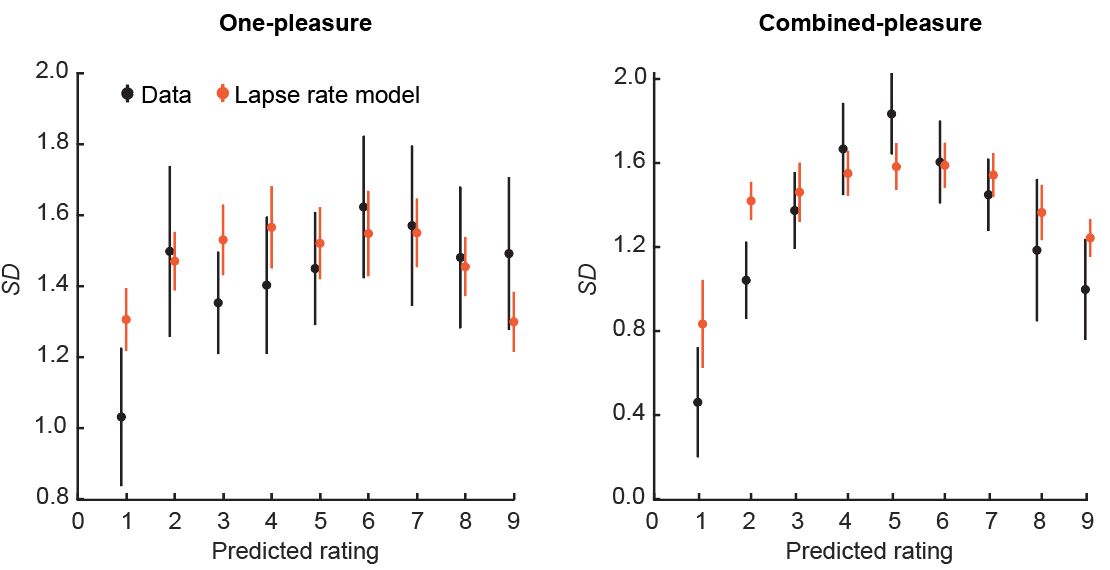


*Figure S10.* Average *SD*s per predicted rating for one- (left) and combined-pleasure trials (right). Predicted ratings refer to the single-image target pleasure for one-pleasure trials and to the average of both images’ single-pleasures for combined-pleasure trials. Data is shown in black, predictions of the lapse rate model in orange. Predictions of the lapse rate model were based on the average of 100 iterations of simulations run separately for each participant using the trial sequence each participant encountered. Error bars represent ±SEM.
